# Causes of hospitalization in systemic lupus erythematosus patients at a tertiary care center: insights from South Asia

**DOI:** 10.1097/MS9.0000000000005708

**Published:** 2026-09-17

**Authors:** Ujjwol Prasad Risal, Shreya Shrestha, Mrikchhya Ghimire

**Affiliations:** a Department of Internal Medicine, B.P. Koirala Institute of Health Sciences, Dharan, Nepal; b Department of Internal Medicine, Civil Service Hospital, Kathmandu, Nepal; c Department of General Practice and Emergency Medicine, B.P. Koirala Institute of Health Sciences, Dharan, Nepal

**Keywords:** hospitalization, lupus flare, SLE, South Asia

## Abstract

**Introduction:**

Patients with systemic lupus erythematosus (SLE) often require hospitalization due to disease flares, infections, or other complications. While studies from high-income countries consistently report flares and infections as leading causes, data from South Asia remain limited. This study aimed to identify the leading causes of hospitalization and describe the associated clinical outcomes of patients admitted to a tertiary care center in Nepal.

**Methods:**

A descriptive cross-sectional study was conducted at the B.P. Koirala Institute of Health Sciences, Nepal, including all eligible SLE patients (aged >16 years and diagnosed for >3 months using the SLICC 2012 criteria) from November 2023 to November 2024. Demographic, clinical, laboratory, and treatment data were collected. Reasons for hospitalization and in-hospital outcomes were recorded. Data were analyzed using descriptive statistics.

**Results:**

Sixty-four patients were included; 93.8% were female, with a mean age of 32.4 ± 11.1 years. SLE flare was the most common cause of hospitalization (56.3%), followed by infection (20.3%). Renal flare accounted for the most flare-related admissions (39.1%), while urinary tract infection and acute undifferentiated fever were the leading infections. Nearly half of the patients (46.9%) were not on any immunosuppressive therapy at admission, and hydroxychloroquine use was suboptimal (67.2%). The mean hospital stay was 5.3 ± 3.0 days. Most patients were discharged in stable condition (95.3%); one required ICU transfer, and one died (1.6%).

**Conclusions:**

Renal flares and infections were the most frequent causes of hospitalization in this South Asian SLE cohort, highlighting the need for early recognition and management.

## Introduction

Patients with systemic lupus erythematosus (SLE) may require frequent hospitalizations due to disease flares, infections, or complications arising from multisystem involvement, leading to substantial morbidity, mortality, and financial burden[1]. Several studies from North America, Europe, and Asia have examined the causes of hospitalization among patients with SLE, consistently identifying disease flares and infections as the leading causes ^[^2–5^]^.

However, data from South Asia are scarce. Hospitalization patterns in this region may differ from those in high-income countries because of differences in infection burden, healthcare access, socioeconomic factors, and treatment practices. Understanding these patterns is essential for improving clinical outcomes, guiding early interventions, and planning in resource-limited settings. Generating such evidence is also essential to guide patient care and healthcare planning in low-resource settings. Although few studies have assessed mortality patterns in patients with SLE in the region,^[^6,7^]^ the causes and outcomes of hospitalization remain poorly characterized.

HIGHLIGHTS

- Hospitalization among patients with systemic lupus erythematosus (SLE) is common.
- There are significant gaps in the literature on the causes of hospitalization in the South Asian region.
- Our study found that renal flares and infections accounted for the majority of hospital admissions among SLE patients.
- Renal flares and infections remain the major causes of hospitalization despite regional differences in disease burden, infection prevalence, and practices.

This study aimed to identify the leading causes of hospitalization and to describe associated clinical outcomes among patients with SLE admitted to a tertiary care center in Nepal. This cross-sectional study was reported in accordance with the STROCSS guidelines[8].

## Methods

### Study design and setting

A descriptive cross-sectional study was conducted in the Department of Internal Medicine at the B.P. Koirala Institute of Health Sciences (BPKIHS), Dharan, Nepal. The study included all eligible patients admitted to the medical ward between 7 November 2023 and 6 November 2024.

### Participants

Patients were eligible for inclusion if they were aged 16 years or older with a diagnosis of SLE for at least 3 months, based on the 2012 Systemic Lupus International Collaborating Clinics (SLICC) criteria[9]. A minimum disease duration of 3 months was selected to reduce potential bias from early presentations, particularly those admitted solely for diagnostic evaluation, such as a renal biopsy. Patients diagnosed within the past 3 months, those younger than 16 years, and those who declined to provide consent were excluded. A consecutive sampling method was applied to include all eligible admissions to the medical ward during the study period.

### Sample size

As this was a hospital-based descriptive study, all eligible patients with SLE admitted during the study period were included. Therefore, no formal sample size calculation was performed.

### Variables

The primary outcome variable was the reason for hospitalization. Additional variables included the following:

- Demographics: age, sex, ethnicity, marital status, and geographic origin.
- Clinical profile: disease duration, comorbidities, and organ system involvement at admission.
- Laboratory parameters: complete blood count, renal and liver function tests, urinalysis, and disease-related biomarkers, as available.
- Treatment details: use of immunosuppressive agents or corticosteroids prior to admission.
- Hospital outcomes: length of stay, discharge status, and in-hospital mortality.
- Flare definition: Flare was defined according to the Lupus Foundation of America consensus, as “a measurable increase in disease activity involving new or worse clinical signs and symptoms and/or laboratory measurements considered clinically significant by the assessor, with usually at least some consideration of a change or an increase in treatment[10].”

### Statistical analysis

Data analysis was carried out using IBM SPSS Statistics (version 26). Continuous variables were summarized as means with standard deviations (SDs) for normally distributed data or medians with ranges for skewed data. Categorical variables were presented as frequencies and percentages.

## Results

A total of 64 patients were included in the study;)60 (93.8%) were female and 4 (6.3%) were male. The mean age of the study population was 32.4±11.1 years (range 15 to 66 years) (Table 1). The most common comorbidities were hypertension (10 patients, 15.6%) and hypothyroidism (8 patients, 12.5%) (Table 2). The mean duration since SLE diagnosis was 61.8 ± 57.6 months. Regarding treatment, hydroxychloroquine (HCQ) was used by 43 patients (67.2%). Among immunosuppressive agents, mycophenolate mofetil (MMF) was the most frequently used (16 patients, 25.0%), followed by azathioprine (11 patients, 17.2%), methotrexate (4 patients, 6.3%), and cyclophosphamide (3 patients, 4.7%). Thirty (46.9%) patients were not receiving any immunosuppressive therapy at the time of admission.

**Table 1:** Demographic characteristics of admitted patients with SLE (*n* = 64).

Parameters	*n* (%)
Mean age (years)	32.4±11.1
Sex	
Female	60 (93.8)
Male	4 (6.3)
Female: male ratio	14.5: 1
Marital status	
Married	44 (68.8)
Unmarried	20 (31.3)
Ethnicity	
Mongol	37 (57.8)
Terai	19 (29.7)
Brahmin/Chhetri	3 (4.7)
Dalit	3 (4.7)
Newar	2 (3.1)
Occupation	
Homemaker	37 (57.8)
Student	14 (21.9)
Shopkeeper	3 (4.7)
Farmer	3 (4.7)
Teacher	2 (3.1)
None	5 (7.8)
Residence	
Hills	48 (75.0)
Plains	16 (25.0)
Smoking status	
Smoker	1 (2.8)
Non-smoker	10 (27.8)
Reformed smoker	24 (66.7)

**Table 2:** Clinical characteristics of patients admitted with SLE (*n* = 64).

Parameters	*n* (%)
Comorbidities	
Hypertension	10 (15.6%)
Hypothyroidism	8 (12.5)
Diabetes mellitus	1 (1.6)
Chronic kidney disease	4 (6.5)
Mean duration of SLE diagnosis in months	61.8 ± 57.6
Mean current prednisolone dose (mg)	10.7 ± 15.7
Mean body mass index (kg/m^2^)	23.5 ± 4.1
Mean systolic blood pressure (mm Hg)	119.7 ± 20.7
Mean diastolic blood pressure (mm Hg)	77.6 ± 15.0
Thrombocytopenia	22 (34.8)
Leukopenia	10 (15.6)
Anemia	54 (84.4)
Lymphopenia	31 (48.4)
Neutropenia	9 (14.1)
Acute kidney injury	19 (29.7)
Hematuria	13 (20.3)
Proteinuria	43 (67.2)
Positive ANA	64 (100.0)
Positive anti-dsDNA	50 (78.1)

SLE flare was the most common reason for hospitalization, observed in 36 patients (56.3%), followed by infection in 13 patients (20.3%) (Fig. 1). Among patients with flares, renal involvement was most frequent (25 patients, 39.1%), followed by hematological (6, 9.4%) and neurological flares (3, 4.7%). The leading causes of infection-related hospitalizations were acute undifferentiated fever and urinary tract infection (Table 3).

**Figure 1.**
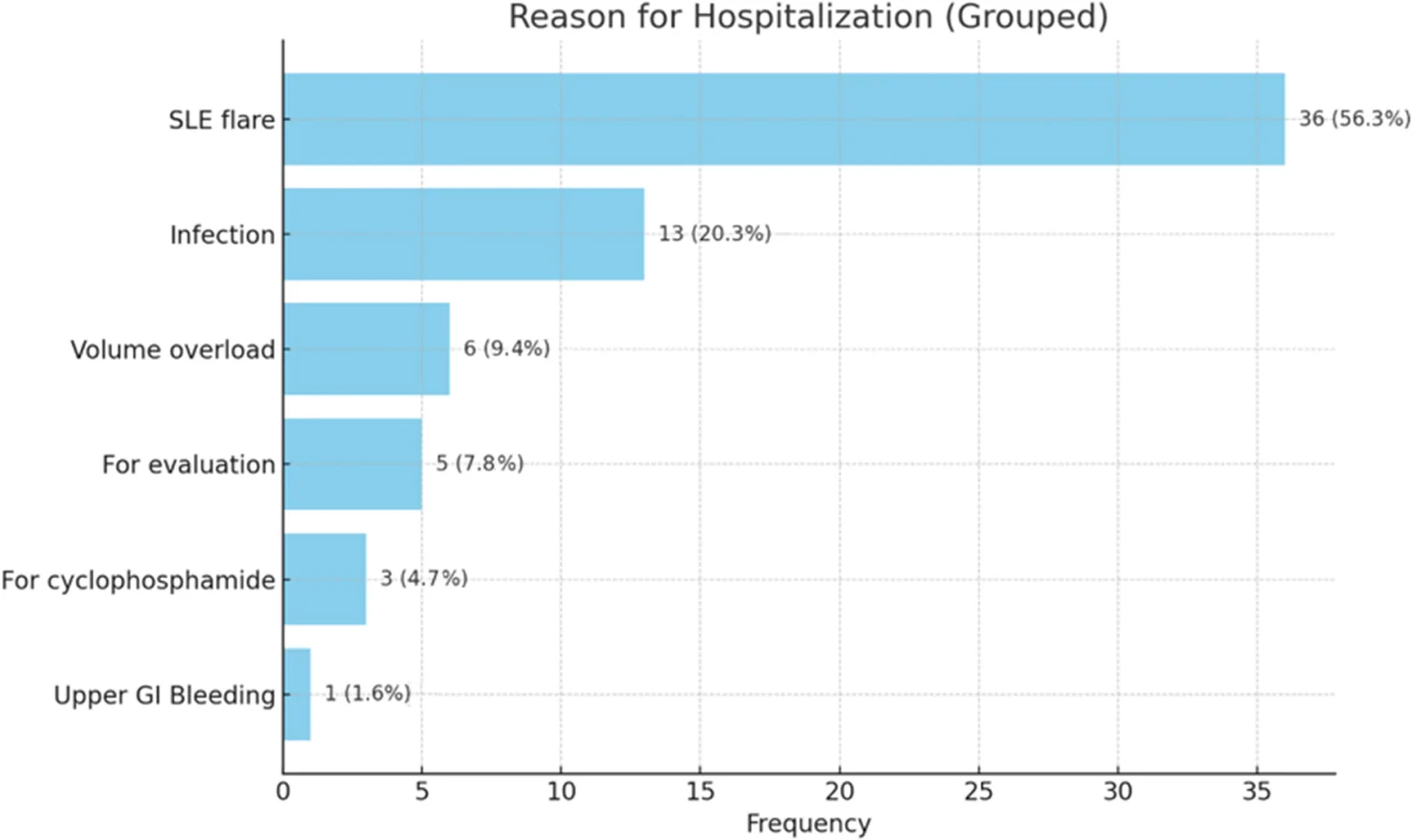
Reasons for hospitalization among patients with SLE.

**Table 3:** Subgroup analysis of various causes of admission (*n* = 64).

Renal flare	25 (39.1%)
Infection	13 (20.3%)
Acute gastroenteritis	2
Pneumonia	2
Urinary tract infection	3
Abscess	2
Acute undifferentiated fever	3
Jugular catheter-related infection	1
Neurological flare	3 (4.7%)
Stroke	2
Peripheral neuropathy	1
Hematological flare (total)	6 (9.4%)
Thrombocytopenia	4
Anemia	2
Musculoskeletal flare	1 (1.6%)
Serositis	1 (1.6%)
Volume overload	6 (9.4%)
For cyclophosphamide	3 (4.7%)
Upper GI bleeding	1 (1.6%)
Evaluation	5 (7.8%)

Eight patients were readmitted twice, and one was readmitted three times. The mean duration of hospital stay was 5.3 ± 3.0 days. Regarding clinical outcomes, 61 patients (95.3%) were discharged in stable condition, 1 (1.6%) required intensive care unit (ICU) transfer, 1 (1.6%) left against medical advice, and 1 (1.6%) died.

## Discussion

This study provides the first detailed analysis from Nepal examining the reasons for hospitalization among SLE patients. Our findings reveal that disease flares and infections were the leading causes of hospitalization, accounting for three-quarters of admissions. These results are consistent with previous studies conducted in other regions, suggesting that despite geographical and resource-based differences, the patterns of hospitalization among SLE patients show notable similarities globally^[^3–5,11–13^]^. In neighboring India, although most studies have focused on mortality rather than hospitalization, comparable findings have been reported[7]. In contrast, cardiovascular manifestations have been reported as a common cause of hospitalization in studies from high-income countries such as the United States^[^2,14^]^. This discrepancy may be attributable to differences in patient populations, healthcare access, and study design, particularly the longer follow-up durations in those cohorts.

Renal flare was the most common cause of hospitalization (39.1%), highlighting the substantial impact of lupus nephritis (LN) in this population. Similar findings have been reported globally, identifying renal involvement as one of the strongest drivers of hospital admission in SLE^[^3,12,15,16^]^. The high frequency of renal flares in our cohort may be attributed to delayed diagnosis, suboptimal adherence to immunosuppressive therapy, or limited access to regular monitoring in low-resource settings. Notably, 46.9% of patients were not on immunosuppressants at the time of admission. In contrast, studies from China and South Korea have reported hematological manifestations as the most frequent reason for hospitalization (14.6% and 30.1%, respectively), followed by renal flares, suggesting regional variability in disease expression and healthcare access^[^5,17^]^. Similarly, in a Tunisian cohort of patients admitted with active disease, hematological and cutaneous manifestations were the most common, followed by active lupus nephritis[18]. In our study, hematological complications (9.4%) represented the second most common cause of flare-driven admission after renal involvement. These included patients with severe thrombocytopenia requiring pulse steroid therapy and those with severe anemia requiring blood transfusion.

Infection was the second most common cause of hospitalization (20.3%), with urinary tract infection, acute undifferentiated fever, and pneumonia being the leading contributors. This highlights the dual challenge faced by clinicians managing SLE: while immunosuppressive therapies are essential to control disease activity, they also predispose patients to serious infections. Similar patterns have been observed in international studies where infections are frequently implicated in both morbidity and mortality among SLE patients^[^2,14^]^. Across Asia, infections consistently emerge as a leading driver of hospitalization and mortality in SLE, though the relative burden varies across regions^[^5–7,15,17^]^.

In our cohort, the susceptibility to infections may also reflect factors such as poor sanitation, delayed medical attention, and suboptimal vaccination coverage.

Volume overload requiring dialysis support was an important cause of hospitalization. Although not indicative of a flare, it reflects accumulated organ damage due to LN progression to chronic kidney disease. This underscores the need for timely diagnosis and aggressive management of LN, along with improved access to long-term care. It also highlights the likelihood that many patients are not receiving adequate or continuous treatment, contributing to preventable disease progression.

Neuropsychiatric manifestations, musculoskeletal involvement, and serositis were other notable reasons for hospitalization. While less common, these complications further emphasize the multisystemic nature of SLE and the need for a multidisciplinary approach to patient management. Although musculoskeletal flares were less frequent in our cohort, studies from Saudi Arabia and Africa reported them as the primary reason for flare-driven admissions^[^15,19^]^. Neurological flares (4.7%) included stroke and peripheral neuropathy, both of which carry substantial implications for long-term disability. Of note, 4.7% of patients required hospitalization exclusively for cyclophosphamide administration, underscoring difficulties in accessing proper care and geographic constraints.

Although other studies have reported thromboembolic events, pregnancy-related morbidity, and adverse drug reactions, we did not encounter such cases at admission^[^2–5^]^. The average length of hospital stay in our study was 5.3 ± 3.0 days, which is shorter than some international cohorts^[^4,12,18,20^]^. This may reflect differences in hospital protocols, resource availability, or the threshold for admission and discharge. Notably, our findings are consistent with durations reported in other studies^[^16,21^]^. Outcomes were generally favorable, with a low in-hospital mortality rate (1.6%), consistent with tertiary care data from other regions^[^2,14^]^. However, the study does not capture post-discharge mortality.

Importantly, HCQ use was suboptimal (67.2%), despite strong evidence supporting its role in preventing flares, reducing organ damage, lowering thrombotic risk, and improving survival[22]. Its suboptimal use may therefore contribute to higher disease activity and hospitalization rates in our cohort. Regarding immunosuppressive therapy, the most commonly prescribed agent was mycophenolate mofetil (25.0%), followed by azathioprine (17.2%), while 46.9% of patients were not on any immunosuppressive therapy. The relatively low percentage of patients on immunosuppressants may be linked to varying disease severity, contraindications, financial constraints, or poor adherence. Given the well-established role of HCQ and immunosuppressive therapies, particularly in LN for preventing flares[22], strategies to improve adherence and ensure wider use of HCQ could significantly reduce hospitalizations in future cohorts.

Our study adds valuable data to the limited body of literature on SLE hospitalizations in Nepal. Compared to prior studies from the country that have primarily focused on clinical profiles or outpatient presentations^[^23,24^]^. this work provides insight into the acute complications and inpatient burden of SLE in a real-world setting.

However, several limitations must be acknowledged. The study was conducted at a single center with a relatively small sample size, which may limit the generalizability of our findings. Although patients diagnosed with SLE within the preceding 3 months were excluded to minimize admissions related to initial diagnostic evaluation and stabilization, the use of a shorter cut-off than the 6-month threshold employed in some LN studies may not have fully eliminated potential lead-time and misclassification bias. SLE disease activity indices were not used as measures of disease activity, which is another limitation. Additionally, due to the cross-sectional design, we were unable to assess long-term outcomes or identify predictors of hospitalization or mortality.

Despite these limitations, our findings highlight the need for improved disease monitoring, early initiation of and adherence to immunosuppressive therapy, and infection prevention strategies in SLE care. Multidisciplinary approaches and stronger outpatient follow-up systems may reduce preventable hospitalizations. Future prospective multicenter studies are needed to explore risk factors for hospitalization and long-term outcomes among patients with SLE in low-resource settings such as Nepal.

## Conclusions

SLE flares, particularly renal involvement and infections, were the leading causes of hospitalization in this South Asian cohort. Suboptimal use of HCQ and immunosuppressive therapy, along with limited access to regular monitoring, may contribute to preventable disease activity and complications. Strengthening early diagnosis, improving treatment adherence, and enhancing preventive strategies are essential to reduce hospitalizations and improve outcomes. Larger multicenter studies are needed to better characterize risk factors and guide interventions in resource-limited settings.

## Data Availability

Datasets generated during the study are available upon request.

## Research registration unique identifying number (UIN)

Not applicable. This prospective observational study was not prospectively registered.

## Declarations

A preliminary version of this work was presented in 2025 at the 16th International Congress on Systemic Lupus Erythematosus, Toronto, Canada. An abstract related to this study was published in the abstract supplement of *Journal of Rheumatology* (May 2025, 52 (Suppl 1: 224-225). The current manuscript contains the full data and expanded analysis.
